# Retrospective comparison of chloroprocaine and mepivacaine in spinal anesthesia for same-day discharge TKA

**DOI:** 10.1186/s43019-025-00283-4

**Published:** 2025-08-07

**Authors:** Noah Gilreath, Jonathan Liu, Cameron Thomson, Mohammad Daher, Sandi Caus, Harrison Dunn, Valentin Antoci, Thomas Barrett, Eric Cohen

**Affiliations:** 1 University Orthopedics, Inc, 1 Kettle Point Ave, East Providence, RI 02914 USA; 2 Department of Orthopaedics, Brown University, Providence, RI USA; 3 UMass Chan Medical School, Worcester, MA USA

**Keywords:** Length of stay, Same-day discharge, Perioperative outcomes, Short-acting spinal anesthesia, Total knee arthroplasty

## Abstract

**Introduction:**

Chloroprocaine is a rapid, short-acting spinal anesthetic that may facilitate quicker postoperative recovery in total knee arthroplasty (TKA). However, limited literature exists regarding its use in patients undergoing same-day discharge (SDD) TKA. This study evaluated the clinical outcomes and safety of chloroprocaine compared with those of mepivacaine.

**Methods:**

This retrospective study of 178 patients who underwent primary TKA at a single ambulatory surgery center from March 2022 to June 2023 compared chloroprocaine (*n* = 114) and mepivacaine (*n* = 64) spinal anesthesia. Surgical outcomes, including estimated blood loss (EBL), operative time, discharge time, 90-day emergency department visits, and 90-day readmissions, were evaluated. In addition, anesthesia-related complications such as hypotension, bradycardia, urinary retention, and postanesthesia care unit (PACU) outcomes were recorded. Continuous variables between the chloroprocaine and mepivacaine groups were compared via independent sample *t*-tests, whereas categorical variables were analyzed via chi-squared tests. A *p*-value of less than 0.05 was considered statistically significant.

**Results:**

There were no differences in the baseline characteristics between the two groups. The chloroprocaine group had a significantly shorter operative time (73.1 versus 84.9 min, *p* < 0.001) and faster discharge (3.7 versus 4.2 h, *p* < 0.001), with no difference in EBL. Patients in the chloroprocaine group had a lower incidence of urinary retention (2.5 versus 15.6%, *p* = 0.004), a lower proportion of patients who needed a urinary catheter (2.6 versus 14.1%, *p* = 0.004), and fewer postoperative complaints of numbness (19.3 versus 39.1%, *p* = 0.004). There were no differences in surgical complications.

**Conclusions:**

Compared with mepivacaine, chloroprocaine spinal anesthesia for SDD TKA was associated with lower rates of urinary retention, a reduced need for urinary catheterization, fewer neurological complaints in the PACU, and faster discharge times. No differences were observed in surgical outcomes between the groups, and there were no instances of unplanned direct admissions. These results suggest that chloroprocaine can be safely used as a reliable alternative to mepivacaine in outpatient TKA.

## Introduction

In 2018 and 2020, total knee arthroplasty (TKA) and total hip arthroplasty (THA) were removed from the Medicare Inpatient-Only list, leading to an increase in same-day discharge (SDD) arthroplasty procedures. The use of spinal anesthesia has been instrumental in the adoption of SDD after total joint arthroplasty (TJA). Spinal anesthesia offers short-acting, rapid-onset analgesia while avoiding the many risks of general anesthesia, namely, postoperative nausea and vomiting, hypotension, respiratory depression, cardiopulmonary stress, airway manipulation, and delirium [1]. Studies have consistently demonstrated superior pain control, faster recovery, fewer complications, and lower costs with the use of spinal anesthesia than with general anesthesia, making it the ideal choice for TJA and fast-track discharge [2–4].

Despite these advantages, however, spinal anesthesia can cause undesirable side effects, such as prolonged motor blockade, transient neurologic symptoms (TNS), and urinary retention [1]. Since spinal anesthesia has demonstrated higher success rates for SDD than general anesthesia, identifying the safest and most effective type of spinal anesthesia has become increasingly important for surgeons. There are many anesthetic agents to choose from, such as bupivacaine, ropivacaine, prilocaine, mepivacaine, and chloroprocaine; each has its own efficacy, safety, onset, and duration of action profiles, and the choice of anesthetic appears to play a large role in discharge readiness after TJA. Bupivacaine, the most widely used spinal anesthetic owing to its low side effect profile, has a long and unpredictable duration of action of 3–9 h, which can create challenges for fast-track discharge [5]. Alternatively, mepivacaine, an anesthetic that gained popularity in the 1980s but fell out of favor owing to its risk of transient neurologic symptoms, has recently been shown to lead to faster return of motor function without any increased adverse events compared with bupivacaine [6, 7].

Chloroprocaine, a rapid-onset, short-acting anesthetic that was similarly pulled from the market in the 1980s owing to the risk of neurologic injury with a 3% formulation, has also recently had a re-emergence [8, 9]. Unlike bupivacaine or mepivacaine, which are amide-type anesthetics, chloroprocaine is an ester-type anesthetic, which contributes to its shorter duration of action [10]. The rapid metabolism of chloroprocaine by plasma cholinesterase, along with its lower lipophilicity and reduced protein binding, further differentiates it from bupivacaine and mepivacaine [11].

There are few studies on the use of chloroprocaine in TKA, but early findings in arthroplasty have shown promising results. A case series involving patients who underwent TKA or THA with chloroprocaine reported a low incidence of nausea and urinary retention, indicating its potential as a safe option for spinal anesthesia in TJA [12]. In addition, studies on chloroprocaine use in THA have demonstrated several advantages over bupivacaine, such as reduced operative times, less intraoperative hypotension, lower estimated blood loss (EBL), and a shorter hospital length of stay (LOS) [13, 14].

To our knowledge, chloroprocaine has not been compared with mepivacaine in the outpatient TKA setting. Here, we compared the outcomes of patients who underwent TKA with those who underwent planned SDD and who received spinal anesthesia with chloroprocaine or mepivacaine. We hypothesize that chloroprocaine can serve as a safe alternative to mepivacaine, leading to reduced time to discharge while achieving fewer or equal undesired side effects and complications.

## Methods

### Case selection

After approval from our institution’s ethical review board (ID no. 1803063) and in accordance with the ethical standards of the Declaration of Helsinki, the records were retrospectively reviewed for all patients who underwent primary TKA by three independent surgeons at the same ambulatory surgical center between March 2022 and June 2023. All TKAs were performed via a standard medial parapatellar approach.

All patients received standard dosing of intravenous tranexamic acid (TXA) intraoperatively, as per the institutional protocol. Postoperatively, all patients underwent the same standardized multimodal pain management and rehabilitation protocol. The postoperative pain management protocol included alternating acetaminophen 975 mg and ibuprofen 600–800 mg, each administered three times daily for 30 days, along with a short course of oxycodone 5 mg, prescribed as 20 tablets total.

Patients were divided into two cohorts on the basis of whether they received chloroprocaine or mepivacaine. The inclusion criteria were patients who were over 18 years old and who had undergone primary TKA with either chloroprocaine or mepivacaine. Patients who received general anesthesia were excluded. For spinal anesthesia, 3% chloroprocaine was used, while either 1.5% or 2% mepivacaine solutions were administered.

The selection of spinal anesthetic is typically determined through collaboration between anesthesia providers and the attending surgeon. Traditionally, mepivacaine was the standard of choice at our institution, with anesthesia providers adjusting the dosage on the basis of body mass index (BMI) and other patient-specific characteristics. From March 2022 to September 2022, mepivacaine was primarily used. The organization transitioned to chloroprocaine beginning in September 2022 through June 2023. This change represented a systemic transition in anesthetic preference rather than a decision influenced by patient-specific demographics.

### Data collection

The collected data included baseline demographics and surgical characteristics, such as the type of spinal anesthesia, EBL, and operative time (elapsed time from incision time to wound closure). Anesthesia-related complications, including hypotension (i.e., mean arterial pressure (MAP) < 65 mmHg), bradycardia (heart rate < 60 beats per minute), and urinary retention. Urinary retention was assessed per protocol by a bladder scan performed immediately postoperatively in the operating room: post-void residual (PVR) volume was measured, and a straight urinary catheter was placed if the PVR exceeded 350 mL. Postanesthesia care unit (PACU) outcomes were also recorded. In addition, postoperative surgical outcomes, such as discharge time (defined as the elapsed time between arrival to PACU and time of discharge), complications, and 90-day readmissions or emergency department (ED) visits, were recorded. Discharge criteria included the ability to tolerate oral intake, safely ambulate with physical therapy following achievement of a Bromage stage 0 motor block, maintain stable vital signs, and independently void.

### Baseline characteristics

There were 178 patients who underwent SDD TKA under spinal anesthesia. Among them, 114 were in the chloroprocaine group, and 64 were in the mepivacaine group. No significant differences were detected in terms of age, sex, side of surgery, BMI, American Society of Anesthesiologists (ASA) classification, or Charlson Comorbidity Index (CCI).

### Statistical analysis

The data were analyzed via SPSS 25.0 software (SPSS Inc., Chicago, IL, USA). Independent samples *t*-tests were performed to compare continuous variables between the chloroprocaine and mepivacaine groups, whereas chi-squared tests were used for categorical variables. A *p*-value of less than 0.05 was considered statistically significant.

## Results

### Surgical characteristics

In terms of surgical characteristics, the chloroprocaine group had shorter operative times (73.1 ± 19.2 versus 84.9 ± 18.3 min, *p* < 0.001) and faster discharge times (3.7 ± 0.9 versus 4.2 ± 0.8 h, *p* < 0.001), with no difference in EBL (Table 1). However, the chloroprocaine group received higher doses of spinal anesthesia than the mepivacaine group (58.4 ± 7.0 versus 51.0 ± 6.9 mg, *p* < 0.001).

**Table 1:** Baseline and surgical characteristics of patients undergoing SDD TKA

Variables	Chloroprocaine (*n* = 114)	Mepivacaine (*n* = 64)	*P*-value
Age (years)^†^	65.2 ± 8.1	63.2 ± 6.1	0.06
Females/males^‡^	59/55	34/30	0.86
Left/right^‡^	54/60	25/39	0.28
BMI^†^	29.7 ± 4.3	29.7 ± 3.6	0.97
ASA score^†^	2.0 ± 0.3	2.0 ± 0.1	0.77
CCI^†^	2.5 ± 1.0	2.1 ± 1.0	0.06
Dose (mg)^†^	58.4 ± 7.0	51.0 ± 6.9	** < 0.001**
EBL (mL)^†^	86.3 ± 39.2	88.9 ± 32.4	0.65
Operative time (min)^†^	73.1 ± 19.2	84.9 ± 18.3	** < 0.001**
Discharge time (h)^†^	3.7 ± 0.9	4.2 ± 0.8	** < 0.001**

^†^Values are presented as mean ± standard deviation

^‡^Values are presented as counts

SDD, same-day discharge; TKA, total knee arthroplasty; BMI, body mass index; ASA, American Society of Anesthesiologists; CCI, Charlson Comorbidity Index; EBL, estimated blood loss

### Anesthesia complications

There was no difference between the two groups in terms of postoperative bladder scan volume, which was measured per protocol at the end of each case to assist in diagnosing urinary retention. However, patients in the chloroprocaine group had a lower incidence of urinary retention (2.5% versus 15.6%, *p* = 0.004), and a lower proportion of patients needed a urinary catheter (2.6% versus 14.1%, *p* = 0.004) (Table 2).

**Table 2:** Comparisons of anesthesia-related complications

Variables	Chloroprocaine (*n* = 114)	Mepivacaine (*n* = 64)	*P*-value
Urine on bladder scan (mL)^†^	168.3 ± 165.8	221.6 ± 161.4	0.06
Urinary catheterization^‡^	3 (2.6%)	9 (14.1%)	**0.004**
Urinary retention^‡^	4 (3.5%)	10 (15.6%)	**0.004**
Intraoperative MAP (mmHG)^†^	60.2 ± 4.9	61.3 ± 5.3	0.22
PACU MAP (mmHG)^†^	61.4 ± 7.2	71.1 ± 15.5	0.08
Intraoperative hypotension^‡^	71 (62.3%)	44 (68.8%)	0.39
PACU hypotension^‡^	10 (8.8%)	3 (4.7%)	0.32
Intraoperative bradycardia^‡^	48 (42.1%)	27 (42.2%)	0.99
PACU bradycardia^‡^	48 (42.1%)	30 (46.9%)	0.54
PACU numbness^‡^	22 (19.3%)	25 (39.1%)	**0.004**
Nausea^‡^	18 (15.8%)	8 (12.5%)	0.55
Vomiting^‡^	3 (2.6%)	1 (1.6%)	0.64
Headaches^‡^	0 (0%)	1.0 (1.6%)	0.18
Pruritus^‡^	2 (1.8%)	0 (0%)	0.29

^†^Values are presented as mean ± standard deviation

^‡^Values are presented as counts with percentage in parentheses

PACU, post anesthesia care unit; MAP, mean arterial pressure

In terms of the MAP intraoperatively and in the PACU, there was no difference between the two groups in terms of the mean MAP or the incidence of hypotension. Furthermore, there was no difference in the incidence of bradycardia between the two groups (Table 2).

There were no cases of spinal dural punctures requiring a blood patch or sedation persisting in the PACU in either group. Furthermore, there was no difference in the incidence of nausea, vomiting, headache, or pruritus between the two groups. However, fewer patients in the chloroprocaine group reported numbness in the PACU (19.3% versus 39.1%, *p* = 0.004) (Table 2).

### Surgical complications

There were no differences in direct admissions or in 90-day ED visits and readmissions. Similarly, there were no differences in revisions (one revision in each group), prosthetic joint infections, or periprosthetic fractures (Table 3).

**Table 3:** Surgical complications

Variables	Chloroprocaine (*n* = 114)	Mepivacaine (*n =* 64)	*P*-value
Direct admissions	0 (0%)	0 (0%)	–
90-day readmissions	4 (3.5%)	0 (0%)	0.13
90-day emergency department visits	9 (8.0%)	5 (7.8%)	0.97
Revisions	1 (0.9%)	1 (1.6%)	0.68
Prosthetic joint infections	0 (0%)	0 (0%)	–
Periprosthetic fractures	0 (0%)	0 (0%)	–

All values are presented as counts with percent in parentheses

The revision in the chloroprocaine group was performed owing to increasing knee pain, instability, and symptomatic popliteus snapping affecting the patient’s quality of life. In the mepivacaine group, the revision involved patellar component exchange due to patellar dislodgement. The four 90-day readmissions in the chloroprocaine group were due to the following causes: one patient was admitted for intraventricular hemorrhage and stroke; another for cardiac arrest and ST-elevation myocardial infarction; a third for a syncopal episode secondary to anemia from an underlying duodenal ulcer; and the fourth for a gastrointestinal bleed attributed to nonsteroidal anti-inflammatory drug (NSAID) use and a bleeding gastric ulcer. There were no 90-day readmissions for the mepivacaine group.

## Discussion

In this study, we compared outcomes between two popular short-acting spinal anesthetic agents, chloroprocaine and mepivacaine, in patients who underwent outpatient primary TKA. We found that patients who received chloroprocaine were discharged faster and had shorter operative times than those who received mepivacaine. Patients in the chloroprocaine group also experienced lower rates of urinary retention, fewer instances of urinary catheterization, and fewer complaints of numbness in the PACU than did those in the mepivacaine group. Both agents were comparable in terms of postoperative surgical outcomes. While prior studies, such as those by Gebhardt et al., have examined these anesthetics in arthroscopic knee procedures, their findings may not be generalizable to the TKA population [15]. To our knowledge, this is the largest study evaluating chloroprocaine use in TKA and the first to directly compare it with mepivacaine in this context. Our findings of faster discharge with chloroprocaine were supported by Gebhardt et al., who reported that patients who received chloroprocaine spinal anesthetic were ready for discharge 44 min faster than those who received mepivacaine or prilocaine [15]. Similarly, randomized control trials by Lacasse et al. and Camponovo et al. demonstrated that compared with bupivacaine, chloroprocaine led to faster recovery and discharge readiness in outpatient orthopedic and abdominal procedures [16, 17].

We also observed that the chloroprocaine group had shorter operative times than the mepivacaine group. Specifically, patients who underwent TKA with chloroprocaine anesthesia had an average operative time that was 11.8 min shorter (*p* < 0.001) than that of those who received mepivacaine. This aligns with findings by Herndon et al., who, in a retrospective review comparing chloroprocaine with bupivacaine in THA, reported a shorter operative time (68.2 versus 83.6 min) with chloroprocaine [14]. While this difference may raise concerns about the impact of shorter-acting anesthetics on surgical pacing, it is important to note that chloroprocaine doses are adjusted by anesthesiologists to provide sufficient anesthetic duration for the anticipated length of surgery. This is reflected in our study, where the chloroprocaine group received higher average doses than the mepivacaine group, in line with standard dosing practices to ensure a comparable intraoperative anesthetic window. However, without head-to-head efficacy equivalence data, comparisons based solely on dose must be interpreted with caution.

The primary purpose of using chloroprocaine is not to shorten the intraoperative window but rather to facilitate more rapid postoperative recovery. The observed shorter operative times in the chloroprocaine group are likely multifactorial and not solely attributable to anesthetic choice. Factors such as surgical efficiency, team workflows, and possibly patient selection may contribute. Although prior studies have speculated that heightened urgency due to chloroprocaine’s shorter duration may lead to reduced teaching or accelerated operative tempo, all surgeries in our study were performed by senior attending surgeons with over 10 years of experience [15]. Moreover, no significant differences were observed between groups in terms of age, gender, BMI, ASA classification, or CCI. While mepivacaine was initially the standard spinal anesthetic at our institution, a shift toward chloroprocaine use occurred institution-wide without patient-level selection. Thus, our findings may reflect increased system-wide efficiency over time, rather than a direct causal effect of the anesthetic alone.

Spinal anesthesia is known to be associated with postoperative urinary retention. In our study, the chloroprocaine group exhibited significantly lower rates of urinary retention (3.5% versus 15.6%) and need for straight urinary catheterization (2.6% versus 14.1%) than the mepivacaine group. The rates of postoperative straight urinary catheterization with mepivacaine have been reported to range from 0% to 10% [6, 18–20]. Conversely, urinary retention rates requiring catheterization with chloroprocaine have been reported to range from 0% to 4% [21–25]. This range for chloroprocaine is significantly lower than the urinary retention rates previously reported with bupivacaine, which are as high as 16.5% [26]. These findings highlight the advantages of chloroprocaine over mepivacaine and bupivacaine in TKA, including reduced anesthesia-related complications and faster recovery.

TNSs are undesirable side effects characterized by temporary lower extremity pain in the absence of identifiable substantial nerve injury or radiologic abnormalities [6]. It typically occurs within 24 h of spinal anesthesia and resolves within 2–5 days [27]. TNS has been associated with longer-acting anesthetics such as bupivacaine and intermediate-acting anesthetics such as lidocaine and mepivacaine. TNS is linked to increased postoperative pain, increased analgesic use, and lower patient satisfaction [28]. In our study, we did not observe any true TNS cases, as screening for these symptoms was performed prior to discharge. However, we noted a significant difference in complaints of numbness documented in the PACU, with fewer complaints among chloroprocaine patients (19.3%) than among mepivacaine patients (39.1%). This difference is likely due to the shorter duration of chloroprocaine. These findings align with prior studies showing a 0% incidence of TNS with chloroprocaine [17, 21, 22, 25].

With respect to postoperative surgical complications, including direct admissions, 90-day readmissions, 90-day ED visits, revision surgeries, prosthetic joint infections, and periprosthetic fractures, there were no significant associations with the type of spinals, and there were very few surgical complications overall in this cohort of patients.

There are some limitations in this study inherent to its retrospective design, including the risk of selection bias. In addition, all 178 TKA procedures were performed by three fellowship-trained arthroplasty surgeons at a single ambulatory surgery center. Although this may have minimized differences in surgical techniques and operating room protocols across institutions, any remaining institutional variations are expected to be minor and unlikely to influence the overall results. While BMI was comparable between groups, the chloroprocaine group received a higher average dose of spinal anesthetic than the mepivacaine group (58.4 ± 7.0 mg versus 51.0 ± 6.9 mg). This discrepancy reflects standard dosing practices, as chloroprocaine is less potent and shorter-acting than mepivacaine and therefore requires a slightly higher milligram dose to achieve a comparable anesthetic effect and is not expected to meaningfully impact outcomes such as block quality or resolution. Anesthesiologists determine dosing on the basis of weight, patient-specific factors, and anticipated surgical complexity and duration, in collaboration with the surgical team. However, as spinal anesthetic dosing was not controlled for in our analysis, we acknowledge this as a limitation of the study. In addition, a post hoc power analysis was not performed, which may limit the ability to fully assess the statistical strength of the observed findings. This represents an area for consideration in future investigations. Nevertheless, our study includes the largest cohort of chloroprocaine use in TKA to date and is the first to directly compare chloroprocaine with mepivacaine in this patient population. Future research with larger sample sizes, a prospective randomized design, and comparisons of a broader range of spinal anesthetics could expand on these findings.

## Conclusions

Compared with mepivacaine, chloroprocaine spinal anesthesia for primary TKA in the setting of SDD resulted in a faster time to discharge, lower rates of urinary retention, a reduced need for urinary catheterization, and fewer complaints of postoperative numbness. There were no differences in terms of surgical outcomes between the groups and no cases of unplanned direct admissions. These findings suggest that chloroprocaine can be safely used as a reliable alternative to mepivacaine in outpatient TKA.

## Data Availability

Data available on request from the authors.
